# Influence of Diet on the Effect of the Probiotic *Lactobacillus paracasei* in Rats Suffering From Allergic Asthma

**DOI:** 10.3389/fmicb.2021.737622

**Published:** 2021-09-27

**Authors:** Ao Xie, Jiaping Song, Shan Lu, Yinhui Liu, Li Tang, Shu Wen

**Affiliations:** Department of Microecology, College of Basic Medical Sciences, Dalian Medical University, Dalian, China

**Keywords:** diet pattern, intestinal microbiota, *Lactobacillus paracasei*, allergic asthma, airway inflammation

## Abstract

Mounting evidence suggests that probiotics can be used to treat allergic asthma by modulating the gut microbiota, and that the effects of probiotics may be influenced by environmental factors such as diet. We conducted a rat model with allergic asthma (AA) modulated by *Lactobacillus paracasei*, feeding up with high-fat or high-fiber diets based on collecting data from 85 questionnaires. The systemic proinflammatory cytokines were detected by ELISA and the overall structure of fecal microbiota was analyzed *via* 16S rRNA gene sequencing. The results showed consumption of a high-fiber diet alleviated the allergic symptoms and airway inflammation, and led to improving the imbalance of T-helper type 1 (Th1)/Th2 cells with increased expression of interferon-γ and decreased expression of interleukin-4. Whereas, the high-fat diet had deteriorating implications and skewed the inflammatory perturbation. Furthermore, abundances of phylum Bacteroidetes, families Muribaculaceae, Tannerellaceae, Prevotellaceae, Enterococcaceae, genera Allobaculum, Parabacteroides, and Enterococcus were enriched in *L*. *paracasei*-modulating rats fed with high-fiber diet. Firmicutes and Proteobacteria, families Lachnospiraceae, Ruminococcaceae and Desulfovibrionaceae, genera Blautia, unidentified_Ruminococcaceae, unidentified_Clostridiales and Oscillibacter were in relatively high abundance in the rats administered high-fat diet. Association between changed microbiota and inflammatory cytokines was also conferred. These data indicated that the efficacy of *L*. *paracasei* in allergic asthma was influenced by different dietary patterns. Hence, diet is important for probiotic therapy when managing allergic asthma.

## Introduction

There has been an increase in the prevalence of allergic asthma (AA) globally, particularly in children and in Western countries, which has led to substantial financial and medical burdens (Colombo et al., 2019). The recommended resolution for AA is to use high-dose inhaled corticosteroids (ICs) alone or combine them with long-acting bronchodilators. Unfortunately, besides the undesirable effects of long-term treatment with ICs, a significant number of asthmatic patients fail to respond to IC therapy.

A disturbed balance of Th1/Th2 cells and Th17/T-regulatory cells (T_*regs*_) imbalance in AA have been reported. Th2 cytokines [e.g., interleukin(IL)-4, IL-5, and IL-13] and potent proinflammatory cytokines (e.g., IL-1β, IL-6, IL-17, IL-25, and tumor necrosis factor (TNF)-α) orchestrate mucosal inflammation. Inflammatory mediators have been shown to impair or boost mucosal inflammation in the airways. Over recent decades, increased perturbation of the microbiota has been demonstrated to contribute to the development of allergic inflammation. Mounting evidence suggests that low diversity of the gut microbiota (GM) in early infancy is a vital risk factor for the development of immune-mediated allergic diseases (Abrahamsson et al., 2014; Tsabouri et al., 2014; Dzidic et al., 2017). Furthermore, microbial colonization in germ-free mice within the first days of life or in antibiotic-treated mice has been indicated to protect against increased IgE levels (Herbst et al., 2011) and promote tolerance to aeroallergens *via* T_*regs*_ induction (Gollwitzer et al., 2014).

Beneficial bacteria modulate the intestinal microbiota and mucosal immune responses. A significantly decreased profile of Th2 cytokines in response to probiotic treatment has been found in animal experiments (Choi et al., 2018; Makino et al., 2019). Oral administration of mixed strains of *Clostridium* species to BALB/c mice has been shown to stimulate the allergen-induced expansion of T_*regs*_ in the colonic mucosa and decreased systemic production of IgE (Atarashi et al., 2011). *Lactobacillus paracasei*, commonly used in dairy-product fermentation, has been shown to mitigate respiratory tract allergies in various studies applying in mice and humans (Fujiwara et al., 2004; Schabussova et al., 2012; Wang et al., 2017; Lin et al., 2020). However, clinical trials have conflicting results in assessing *L*. *paracasei* prevents allergy (Kuitunen et al., 2009; Fiocchi et al., 2012; Jensen et al., 2012; West et al., 2013).

Several studies have underlined the critical role of diet in AA pathogenesis. High consumption of fat (e.g., Western diet) is likely related to an increased risk of asthma (Hou et al., 2011; Protudjer et al., 2012; Manzel et al., 2014; Myles, 2014; Thorburn et al., 2014; Julia et al., 2015). Diets high in fiber (e.g., fruit and vegetables) protect from allergies and are associated with decreased inflammation in the airways (Ellwood et al., 2001; Maslowski et al., 2009; Frei et al., 2012; Grimshaw et al., 2014; Trompette et al., 2014). The effect of diets on allergic asthma might have a close association with the intestinal microbiota. Different diets yield numerous metabolites that can influence the immune response, such as short-chain fatty acids (SCFAs) fermented by colonic commensal, responsible for the effect of anti-inflammation, and the saturated fatty acids or cholesterol displayed exacerbation for allergic disease. However, how to promote the effect of probiotics in people with different diets is not known.

Herein, we collected data (*via* questionnaires) from 85 human participants to design specific different dietary patterns and established a rat model of AA (using ovalbumin[OVA]) fed up with the designed diets to assess the efficacy of different diets on *L*. *paracasei*-modulated allergic inflammation. Our findings emphasize the importance of diet to explain the effect of *L*. *paracasei* on AA prevention.

## Materials and Methods

### Collection of Dietary Data

Collection of dietary data was conducted from 25 November to 2 December 2017. The food inventory was documented according to the Chinese Dietary Guidelines (fourth edition, 2016) and AA history. Food (including edible oils and salt) consumption was determined by weighing, individuals aged >18 years, and excluding those unable to complete data collection.

### Preparation of Probiotics

*Lactobacillus paracasei* DMLA16017 (Department of Microecology, Dalian Medical University) was grown in MRS broth (Hopebio, Qingdao, China) at pH 5.7 ± 0.2 and under anaerobic conditions. Bacteria were harvested in the early stationary phase, washed with phosphate-buffered saline (PBS; Solarbio, Beijing, China), and stored at −80°C. Cell counts were determined by plating serial dilutions.

### Animals and Intervention Design

The study protocol was approved by the Animal Ethics Committee of Dalian Medical University (No. L201312). Specific pathogen-free (SPF) three-week-old male Wistar rats were purchased from the Animal Center of Dalian Medical University and allowed to acclimatize to their surroundings for ≥24 h before experimentation. Rats were housed under standard temperature conditions and humidity with a 12-h light-dark schedule with free access to sterile food and autoclaved water.

**FIGURE 1:**
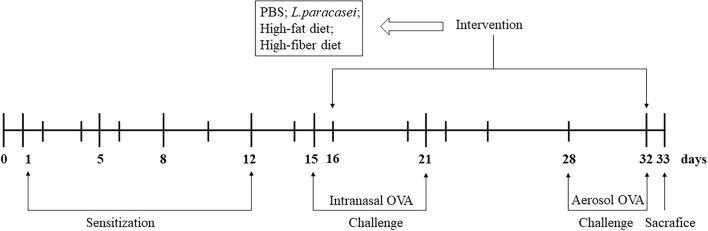
Experimental design of a model of allergic asthma in rats. Male Wistar rats were sensitized and challenged with OVA, as described in “Animals and Intervention Design” section. Groups were allocated as Con, Model, Probio, Hfat, and Hfiber. From day 16 until day 32, rats were treated daily with *Lactobacillus paracasei*, and then randomly assigned to receive basic chow, high-fat chow or high-fiber chow. Rats in the Con group and Model group received PBS only. All rats were sacrificed on day 33 after documentation of allergic symptoms.

An AA model was established according to a modification of airway inflammation described by Zhang and Shi (Zhang et al., 2016). After acclimatization, rats were assigned randomly to five groups (*n* = 8/group): the control group (Con), the model group (Model), the probiotics-modulated group (Probio), the high-fat diet intervented probiotics-modulated group (Hfat), and the high-fiber diet intervented probiotics-modulated group (Hfiber). Sensitizations were undertaken on days 1, 5, 8, and 12 in all groups except the Con group. Rats were sensitized to OVA (egg-white albumin of chickens ≥98%; Sigma-Aldrich, Saint Louis, MO, United States) by intraperitoneal injection of 0.2 mL of alum-precipitated antigen [comprising 10 μg of OVA absorbed into 1 mg of aluminum hydroxide (Sigma-Aldrich)]. From day 15,rats were administered (i.n.) 20 μL of 1% OVA diluted in PBS for 7 consecutive days and exposed daily to 1% OVA aerosol in PBS in an aerosol cabin for 30 min between day 28 and day 32 in the challenge phase. Rats in the Con group received PBS alone (Figure 1).

After developing airway inflammation, other groups except Con and Model group were gavage with 1.0 × 10^9^ colony-forming units of *L*. *paracasei* suspended in 0.2 mL of PBS per day, 1 h before the challenge. Rats that underwent gavage with *L*. *paracasei* were received high-fat (Hfat), high-fiber (Hfiber), or basic chow (Con, Model and Probio), respectively, that we purchased from Huafukang Bioscience (Beijing, China) (see Supplementary Table 1 for nutritional parameters). The intervention was from day 16 until day 32 (Figure 1). Twenty-four hours after the final challenge, all animals were sacrificed.

### Evaluation of Allergy Symptoms

For each rat, the frequency of two symptoms of allergy (nose rubbing and sneezing) was recorded immediately for 15 min after the last challenge with OVA aerosol on day 32 in a blinded manner, as described previously (Shinmei et al., 2009).

### Cell Counts for Bronchoalveolar Lavage Fluid (BALF)

After sacrifice on day 33, lungs were perfused with 1 mL of PBS containing 10% fetal bovine serum through a tracheal cannula to collect BALF. The latter was centrifuged at 1000 × *g* for 10 min at room temperature, and the supernatant was separated and stored until use. The pellet was resuspended in PBS for cell counting. BALF cells contain leukocytes and provide a “snapshot” of airway disease.

### Enzyme-Linked Immunosorbent Assay (ELISA) for Total Level of OVA-Specific IgE and Cytokines

Twenty-four hours after the final challenge with OVA, rats were anesthetized and blood samples were withdrawn by cardiac puncture. Serum was separated by centrifugation (1000 × *g*, 10 min, room temperature) after resting for 1 h. The OVA-specific IgE level in serum was determined with an ELISA kit (No. RX-D302482R) according to manufacturer (Ruixinbio, Quanzhou, China) instructions. Absorbance was measured at 450 nm.

The level of IL-4, IL-17, interferon(IFN)-γ, and TGF-β in BALF and serum prepared as stated above were measured by ELISA kits (No. SEA077Ra, No. SEA063Ra, No. SEA049Ra; and No. RX302048R) following manufacturer (Cloud-Clone, Wuhan, China; Ruixinbio, Quanzhou, China) instructions.

### Histology of Lung Tissue

After the sacrifice of rats on day 33, the left-lung lobes were removed rapidly and fixed in 4% paraformaldehyde for ≥24 h at room temperature. Then, lung tissues were dehydrated in a graded series of ethanol solutions, cleared in xylene, and embedded in paraffin. Histology slices (thickness = 5 μm) were prepared using a microtome. These slices were stained with hematoxylin and eosin (H&E) for morphology evaluation and inflammation using a microscope (BX50; Olympus, Tokyo, Japan) equipped with a digital camera (DFC 320, Leica, Wetzlar, Germany).

### DNA Extraction and 16S rRNA Gene Sequencing of Bacteria

Thirty-five fresh feces from rats (seven from Con, five from Model, seven from Probio, eight from Hfat, and eight from Hfiber groups) were harvested aseptically with 2-mL sterilized Eppendorf tubes, respectively, and stored at −80°C immediately.

DNA isolation and 16S rRNA gene sequencing were provided by Novogene (Beijing, China), as described previously (Wang et al., 2016; Zhang et al., 2018), to ascertain the composition of the bacteria in the feces of rats in different groups. DNA was extracted using the cetyl trimethyl ammonium bromide/sodium dodecyl sulfonate (CTAB/SDS) method. The V3-V4 sequences of 16S rRNA genes were amplified by polymerase chain reaction (PCR) using specific primers (341 forward: 5′-CCTAYGGGRBGCASCAG-3′; 806 reverse: 5′-GGACTACNNGGGTATCTAAT-3′). The PCR-amplified product was purified with a gel extraction kit (Qiagen, Hilden, Germany). After quantification, amplicons were normalized to generate a sequencing library and sequenced on a NovaSeq^TM^ platform (Illumina, San Diego, CA, United States).

Alpha diversity, including Chao1 and ACE, was analyzed with Quantitative Insights Into Microbial Ecology (QIIME) 1.7.0^1^. Beta diversity was ascertained using the non-metric multi-dimensional scaling (NMDS) of QIIME 1.7.0 to determine the similarity of species diversity in different samples. Statistical significance was evaluated according to stress. Significant differences in the relative abundance of genera among strains were obtained by linear discriminant analysis (LDA) effect size (LEfSe).

### Statistical Analyses

Continuous variables with a normal distribution were represented as the mean ± SEM, and comparisons between groups were determined by the parametric Student’s *t*-test. For parameters with a non-normal distribution, median and interquartile range (IQR) values are provided, and males were compared with females using the non-parametric Wilcoxon rank-sum test. Absolute and relative frequencies were calculated for qualitative data, and tested by the chi-square test or Fisher’s exact test. Pearson’s correlation was computed between intestinal microbiota and cytokines. *p* < 0.05 was considered significant. Missing values were not replaced and did not contribute to the analysis of the variable. Experiments were carried out at least twice. Data were analyzed using Prism 8.0 (GraphPad, San Diego, CA, United States).

### Accession Number

The sequence data from this study are deposited in the GenBank Sequence Read Archive with the accession number SUB9930073.

## Results

### Collection of Dietary Data

The data [age, body mass index (BMI), allergy history, and daily food intake] of 85 study participants [40 (47.1%) men and 45 (52.9%) women] are shown in Table 1. The median (IQR) age at study inclusion was 35 (27-48) years in men and 34 (26-43) years in women. Forty-nine (57.6%) participants reported a normal BMI (18.5-23.9 kg/m^2^).

**TABLE 1:** Characteristics of study participants.

	**All (*n* = 85)**		**Male (*n* = 40)**		**Female (*n* = 45)**		***P*_*for sex*_**
	***n***	**%**	***n***	**%**	***n***	**%**	
**Age (years)**							
18–26	21	24.7	9	42.8	12	57.2	0.532
27–35	27	31.8	13	48.1	14	51.9	0.723
36–44	14	16.5	6	42.9	8	57.1	0.365
45–53	16	18.8	8	50.0	8	50.0	0.332
54–62	7	8.2	4	57.1	3	42.9	0.246
**BMI (kg/m^2^)**							
Underweight (<18.5)	7	8.2	3	42.9	4	57.1	0.691
Normal (18.5–23.9)	49	57.6	15	30.6	34	69.4	0.670
Overweight (24–29.9)	28	32.9	21	75.0	7	25.0	0.417
Obesity (≥30)	1	1.2	1	100.0	0	0.0	—
**Allergic**							
Yes	44	51.8	19	43.2	25	56.8	0.518
No	41	48.2	21	51.2	20	48.8	

Table 2 shows the percentages of major-diet components in study participants. A significant difference was registered in the intake of vegetables, soybeans, animal-based food, and condiments. Excessive intake of soybeans (58.18 ± 49.45 vs. 39.34 ± 34.41 g), animal-based food (meat: 220.77 ± 117.58 vs. 108.51 ± 103.15 g; fish and shrimps: 103.75 ± 73.86 vs. 62.61 ± 33.88 g; eggs: 47.20 ± 29.13 vs. 34.20 ± 25.88 g) and condiments (oil: 38.27 ± 16.29 vs. 23.85 ± 9.36 g; salt: 7.86 ± 2.95 vs. 5.20 ± 1.79 g) was common in people suffering from allergy compared with that in participants not suffering from allergy. An inadequate intake of vegetables was common in people suffering from allergy (319.43 ± 125.15 g) compared with that in participants not suffering from allergy (387.83 ± 124.62 g) (Table 2).

**TABLE 2:** Components of food intake and its percentage in study participants.

**Components**			**Percentage (%)**									***P* value**
**Cereals**	Intake amount (g)		<100	100–175	175–250	250–325	325–400	400–475	475–550	550–625	≥625	
	**Cereals (%)**	**allergic**	0.0	15.9	2.3	29.5	27.3	2.3	13.6	6.8	2.3	0.918
		**non-allergic**	0.0	4.9	0.0	36.6	29.3	14.6	7.3	2.4	4.9	
**Vegetables**	Intake amount (g)		<100	100–200	200–300	300–400	400–500	500–600	≥600			
	**Vegetables (%)**	**allergic**	2.3	18.2	6.8	27.3	38.6	6.8	0.0			**0.014**
		**non-allergic**	2.4	4.9	4.9	29.3	36.6	17.1	4.9			
**Fruits**	Intake amount (g)		<100	100–200	200–300	300–400	400–500	500–600	≥600			
	**Fruits (%)**	**allergic**	13.6	13.6	34.1	25.0	9.1	4.5	0.0			0.779
		**non-allergic**	7.3	17.1	39.0	29.8	2.4	4.9	2.4			
**Soybeans**	Intake amount (g)		<20	20–40	40–60	60–80	≥80					
	**Soybeans (%)**	**allergic**	11.4	15.9	34.1	29.5	9.1					**0.046**
		**non-allergic**	9.8	50.0	25.0	0.0	9.1					
**Animals food**	Intake amount (g)		<50	50–100	100–150	150–200	200–250	250–300	300–350	350–400	≥400	
	**Meat (%)**	**allergic**	4.5	4.5	27.3	0.0	15.9	25.0	11.4	4.5	6.8	**<0.001**
		**non-allergic**	14.6	53.7	12.2	0.0	4.9	4.9	2.4	2.4	4.9	
	Intake amount (g)		<50	50–100	100–150	150–200	≥200					
	**Fish and shrimp (%)**	**allergic**	25.0	15.9	22.7	25.0	11.4					**0.002**
		**non-allergic**	22.0	58.5	19.5	0.0	0.0					
	Intake amount (g)		<25	25–50	50–75	≥75						
	**Egg (%)**	**allergic**	18.2	20.5	52.3	9.1						**0.033**
		**non-allergic**	19.5	68.3	4.9	7.3						
**Condiments**	Intake amount (g)		<25	25–50	≥50							
	**Oil (%)**	**allergic**	11.4	52.3	36.4							**<0.001**
		**non-allergic**	46.3	48.8	4.9							
	Intake amount (g)		<6	6–12	≥12							
	**Salt (%)**	**allergic**	4.5	88.6	6.8							**<0.001**
		**non-allergic**	58.5	41.5	0.0							

*Forty-four out of 85 individuals had suffered from allergy. Significant *p*-values are indicated in bold font.*

### Different Diet Influences the Effect of *L. paracasei* on Allergic Symptoms

**FIGURE 2:**
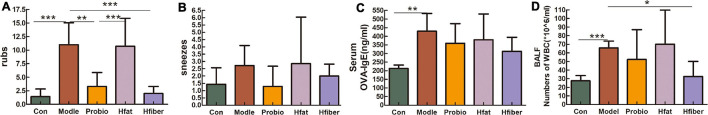
Allergy symptoms resulting from different diets influencing the effect of *L. paracasei*. **(A)** Number of nose rubs. **(B)** Number of sneezes. **(C)** Altered serum level of OVA-specific IgE. **(D)** Absolute number of WBCs in BALF 24 h after final challenge with OVA. Each bar represents the mean ± SEM; *p < 0.05, **p < 0.01, and ***p < 0.001.

To evaluate allergy symptoms in rats, we measured the frequency of nasal rubs and sneezes per rat during the 15 min observation period after the last OVA challenge immediately. Rats in the Model group showed increased nasal rubs (vs. Con group, *p* < 0.001). With probiotics modulation, the number of nasal rubs decreased (vs. Model group, *p* < 0.01). High-fat diet intervention caused a significantly higher number of nasal rubs in probiotics-modulation AA rats (*p* < 0.001), compared with that in the Probio group, along with a reduced nasal rubs in Hfiber group but not significantly (Figures 2A,B). Whereas, the allergic symptoms of rats in Hfiber group was inhibited significantly (vs. Model group, *p* < 0.001). The incidence of sneezes in rats in all the groups maintained no significance. These results indicated that a high-fiber diet acted synergistically with *L*. *paracasei* intervention to alleviate allergic symptoms.

The level of OVA-specific IgE in serum was investigated to elucidate the effect of different diets on *L*. *paracasei* that modulated the allergic response. The serum OVA-specific IgE level was higher in the Model group than in the Con group (*p* < 0.01). There was a trend toward reducing OVA-specific IgE with high-fiber diet treatment (*p* = 0.063), although not significantly (Figure 2C).

We counted the number of inflammatory cells obtained from BALF 24 h after the final challenge with OVA (Figure 2D). An increased total number of white blood cells (WBCs) in BALF was detected in the Model group compared with that in the Con group (*p* < 0.001). Level of WBCs after administered *L*. *paracasei* was lower in Probio group, although this difference was not statistically significant. However, when the rats consumed the high-fiber diet, the total number of inflammatory cells reduced markedly compared with that in the Model group (*p* < 0.05). These data suggested that the total number of WBCs in BALF was inhibited significantly by dietary consumption of fiber, whereas a high-fat diet aggravated the inflammatory response of AA.

### Airway Inflammation in *L*. *paracasei*-Modulating AA Rats Fed Different Diets

**FIGURE 3:**
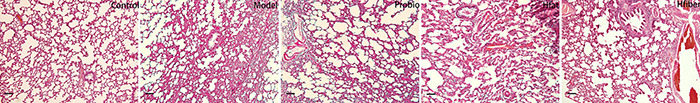
Histology of H&E-stained lung tissue (original magnification: ×100). Photomicrographs were captured to detect eosinophil infiltration. Eosinophil infiltration was significantly higher in the Hfat group than the other groups. Interestingly, eosinophil inflammation was suppressed dramatically consumption of a high-fiber diet. *n* = 5-8 rat/group.

To examine altered inflammation in allergic airways, histological examination of the lung tissue of AA rats was undertaken. Eosinophilic cytoplasm stained red with H&E (Figure 3). The typical pathological features of AA were observed in Model group as compared to Con group, along with discernible damage, edema, and a thickened mucosa. Probiotics treatment was effective in reducing the inflammation. Compared with the Model group, a more robust inflammatory response occurred in the Hfat group: extensive infiltration by eosinophils in peribronchial and alveolar septa, along with markedly thickened alveolar and bronchial walls. However, infiltration of inflammatory cells was suppressed significantly in the Hfiber group.

### Alteration of Cytokine Levels From T Cells by Consumption of Different Diets Influenced *L. paracasei* Intervention

**FIGURE 4:**
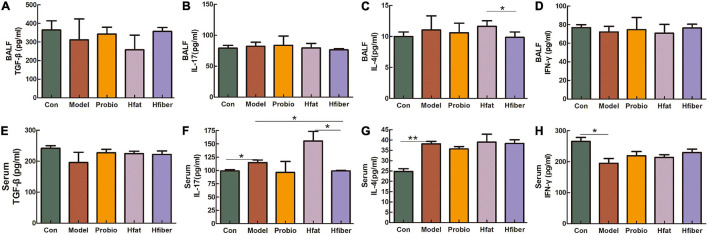
Altered levels of the cytokines after OVA challenge. **(A)** TGF-β in BALF. **(B)** IL-17 in BALF. **(C)** IL-4 in BALF. **(D)** IFN-γ in BALF. **(E)** TGF-β in serum. **(F)** IL-17 in serum. **(G)** IL-4 in serum. **(H)** IFN-γ in serum. Data are the mean secretion (pg/mL) ± SEM, *n* = 4; ^∗^*p* < 0.05, ^∗∗^*p* < 0.001.

We measured cytokine levels in BALF and serum after the OVA challenge to further investigate the skewing of Th1/Th2 and Th17/T_*regs*_ immune responses after consuming different diets and how this influenced *L*. *paracasei* intervention. As shown in Figure 4, discrepancy is apparent in applying the *L*. *paracasei* modulating AA when there are different diets intervented. Notably, compared with the Hfat group, IL-4 was dropped significantly in Hfiber group (*p* < 0.05). The Hfiber group contained apparently lower Th17 cytokine IL-17 level in serum compared with the Model group (*p* < 0.05). Increased production of IL-17 was observed intervented with high-fat diet, compared with Hfiber group (*p* < 0.05). Th1 cytokine IFN-ɤ in both BALF and serum were on the trend opposite from IL-4, although the differences were not statistical significant. The ratio of IL-4/IFN-γ was significantly different in Hfat group from that in Hfiber group (*P* < 0.05) (see Supplementary Figure 1). Based on these results, it was indicated that different diets affect the profile of cytokines in AA during the modulation of probiotics. The high-fiber diet blockaded the allergic inflammation significantly by regulating the levels of multiple inflammatory mediators therein, while the high-fat diet exacerbated it.

### GM Composition of AA Rats Using 16S rRNA Gene Sequencing

A total of 1,915 operational taxonomic units (OTUs) were shared out of sequences obtained from 35 fecal samples. Nineteen phyla, 240 genera, and 162 species of gut microbes were annotated for subsequent analyses.

**FIGURE 5:**
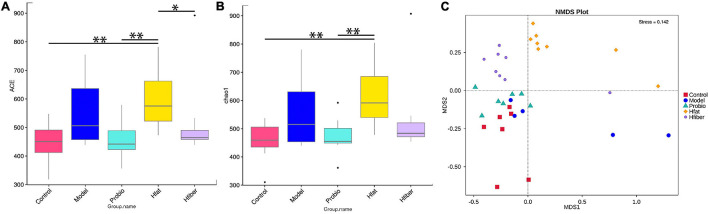
Diversity in microbiota-community structure in different rat groups. **(A)** ACE indice. **(B)** Chao1 indice. *p < 0.05, **p < 0.01. **(C)** NMDS. Stress < 0.2, *p* < 0.05.

We wished to evaluate changes in the structure of the microbiota community among groups fed a different diet. Hence, microbial alpha diversity was measured using ACE and Chao1. As a result, we discovered a significantly higher alpha diversity for the Hfat group compared with that in the Probio group (*p* < 0.01) (Figures 5A,B). Furthermore, ordination with NMDS showed distinct spatial clustering between five groups (stress = 0.142) (Figure 5C).

**FIGURE 6:**
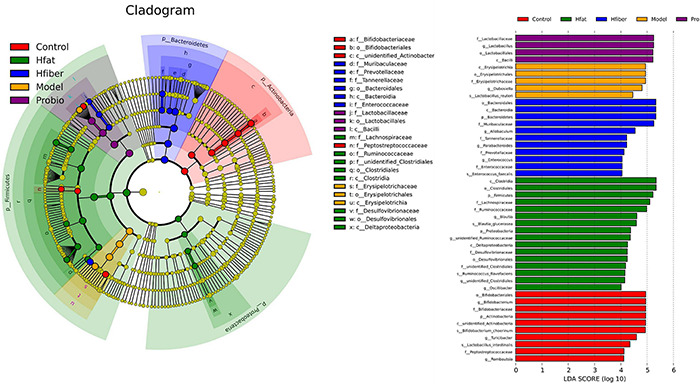
Gut-microbiota comparison analyzed by LEfSe. In the cladogram, changes in the relative abundance of bacterial taxa identified by LEfSe are shown in phylogenetic trees from the phylum to the genus (from the inner to the outer ring, respectively). In the cladogram, the size of the node circle is proportional to the relative abundance of taxa. The node size corresponds to the average relative abundance of the OTU. Yellow indicates no significant difference between groups, and other colors (red, blue, purple, green, and orange) denote that OTUs have a significant difference between groups. Histogram of the LDA scores for differentially abundant genera. LDA scores were calculated by LEfSe using linear discriminant analysis to assess the effect size of each differentially abundant bacterial taxa. Only taxa meeting LDA > 4 are shown.

To further illustrate the differences in gut microbiota composition among groups, we applied the LEfSe method to discover biomarkers in high-dimensional data and reveal genomic characteristics (Figure 6). Differentially enriched bacterial colonizers with LDA > 4 were identified among the five groups. Phylum Actinobacteria, families Bifidobacteriaceae and Peptostreptococcaceae, genera *Bifidobacterium*, *Turicibacter*, and *Romboutsia* were enriched in Con group. Family Erysipelotrichaceae and genus *Dubosiella* were enriched in Model group. Abundances of family Lactobacillaceae and genus *Lactobacillus* were enriched in Probio group. Abundances of phylum Firmicutes and Proteobacteria, families Lachnospiraceae, Ruminococcaceae and Desulfovibrionaceae, genera *Blautia*, *unidentified*_*Ruminococcaceae*, *unidentified*_*Clostridiales* and *Oscillibacter* were enriched in Hfat group. Biomarkers in Hfiber group including phylum Bacteroidetes, families Muribaculaceae, Tannerellaceae, Prevotellaceae, Enterococcaceae, genera *Allobaculum*, *Parabacteroides*, and *Enterococcus*.

To determine whether specific bacteria were differentially abundant along with the probiotics modulation intervented by different diets, we conducted a further analyses. After the AA model had been established, family Erysipelotrichaceae and genera *Dubosiella* and *Paraprevotella* were more abundant, compared with that in the Con group (see Supplementary Figure 2). After intervention with *L*. *paracasei*, the dominant bacteria changed into phylum Bacteroidetes, and there were significant decrease in family Erysipelotrichaceae and genus *Dubosiella* (see Supplementary Figure 3). Upon consumption of different diets, the intestinal community altered significantly. In the Hfat group, phylum Proteobacteria, families unidentified_Clostridiales, Desulfovibrionaceae, Ruminococcaceae and Lachnospiraceae, and genera *Anaerotruncus*, *Oscillibacter*, *Candidatus*_Soleaferrea and *Blautia* were dominant, while phylum Actinobacteria, families Lactobacillaceae, Bifidobacteriaceae and Muribaculaceae, genera *Lactobacillus*, *Bifidobacterium, and Turicibacter* decreased significantly (see Supplementary Figure 4). In the Hfiber group, the phylum Bacteroidetes, families Tannerellaceae, Lachnospiraceae and Muribaculaceae, genera *Angelakisella*, *Parabacteroides*, and *Allobaculum* were in relatively high abundance, phyla Firmicutes and Actinobacteria, families Lactobacillaceae, Bifidobacteriaceae, and Corynebacteriaceae, genera *Lactobacillus Bifidobacterium*, *Turicibacter*, and *Corynebacterium* decreased (see Supplementary Figure 5). Taken together, these data demonstrated that different diets promoted distinct microbial communities that influenced *L*. *paracasei*-modulated AA.

### Correlation Analysis for the Intestinal Microbiota and Cytokine Expression in BALF

The Pearson correlation coefficients between the predominant intestinal microbiota and cytokines in BALF were displayed in Table 3. The BALF level of IL-4 was positively correlated with the abundance of Lachnospiraceae, Deltaproteobacteria, Desulfovibrionaceae, and Clostridiales, whereas TGF-β was negatively correlated with the abundance of Deltaproteobacteria and Desulfovibrionaceae, respectively (*p* < 0.05). The BALF level of IFN-ɤ was negatively associated with Erysipelotrichales abundance (*r* = −0.483, *p* = 0.031). A strong positive association and high significance between the IL-17 and abundance of *Turicibacter*, Peptostreptococcaceae, and *Romboutsia*, respectively, were found (*p* < 0.05).

**TABLE 3:** Pearson correlation coefficients between intestinal bacteria and cytokines in BALF.

**Intestinal bacteria**	**IL-4(pg/mL)**		**IFN-ɤ(pg/mL)**		**IL-17(pg/mL)**		**TGF-β (pg/mL)**	
	***r***	***P***	***r***	***P***	***r***	***P***	***r***	***P***
Ruminococcaceae	0.393	0.087	–0.021	0.930	0.124	0.602	–0.380	0.098
*Lactobacillus*	–0.232	0.326	0.415	0.069	0.071	0.765	0.062	0.795
*Blautia*	0.078	0.743	–0.157	0.508	–0.183	0.440	–0.113	0.637
Bifidobacteriaceae	–0.158	0.505	0.060	0.801	0.185	0.435	0.402	0.079
***Turicibacter***	0.224	0.343	–0.048	0.840	**0.502**	**0.024**	0.019	0.938
Erysipelotrichaceae	0.055	0.816	–0.483	0.816	–0.018	0.939	0.126	0.597
*Oscillibacter*	0.233	0.323	0.076	0.750	0.084	0.725	–0.205	0.386
**Peptostreptococcaceae**	0.279	0.233	–0.218	0.357	**0.612**	**0.004**	–0.020	0.934
***Romboutsia***	0.211	0.372	–0.127	0.595	**0.632**	**0.003**	0.091	0.701
**Lachnospiraceae**	**0.470**	**0.037**	–0.098	0.681	–0.088	0.712	–0.294	0.208
**Erysipelotrichales**	0.055	0.816	−**0.483**	**0.031**	–0.018	0.939	0.126	0.597
**Deltaproteobacteria**	**0.465**	**0.039**	–0.062	0.794	–0.034	0.887	−**0.527**	**0.017**
*Bifidobacterium*	–0.158	0.505	0.060	0.801	0.185	0.435	0.402	0.079
Enterococcaceae	–0.097	0.684	–0.175	0.461	–0.273	0.244	–0.322	0.166
*Parabacteroides*	–0.325	0.162	0.090	0.705	–0.193	0.414	0.194	0.414
Prevotellaceae	–0.225	0.339	–0.005	0.983	–0.216	0.360	–0.009	0.971
**Clostridiales**	**0.513**	**0.021**	–0.115	0.629	0.070	0.768	–0.394	0.086
Tannerellaceae	–0.325	0.162	0.090	0.705	–0.193	0.414	0.194	0.414
*Allobaculum*	–0.254	0.279	–0.188	0.428	–0.390	0.089	0.229	0.332
Muribaculaceae	–0.334	0.150	0.031	0.897	–0.131	0.583	0.252	0.283
*Dubosiella*	–0.010	0.966	–0.377	0.101	–0.061	0.799	0.081	0.735
Lactobacillaceae	–0.232	0.326	0.415	0.069	0.071	0.765	0.062	0.795
**Desulfovibrionaceae**	**0.478**	**0.033**	–0.050	0.835	–0.026	0.913	−**0.538**	**0.014**
*Enterococcus*	–0.097	0.684	–0.175	0.461	–0.273	0.244	–0.322	0.166
*unidentified_Clostridiales*	0.425	0.061	–0.275	0.240	–0.081	0.732	−**0.583**	**0.007**

*p < 0.05, **p < 0.01. *P* < 0.05 is highlighted in bold.

## Discussion

Diet affects the GM, and dietary habits can impact overall intestinal health (Nakaji et al., 2002; Gophna, 2011; Wu et al., 2011; Bernaud and Rodrigues, 2013; Trompette et al., 2014). However, little is known of the role of diet in probiotic-modulating AA. One might speculate an interplay of diet, intestinal microflora, and immune cells. The diet pattern could be critical in regulating the effect of probiotics given to people suffering from AA.

The dietary patterns of 85 individuals were obtained. We found that people suffering from AA had unbalanced diets and inadequate intake of vegetables, and excessive intake of animal-based food was typical. According to a World Health Report from 2014, low intake of vegetables and fruit is associated with ischemic heart disease, gastrointestinal cancer, and stroke (World Health Organization (WHO), 2014). Consistent with the notion that a plant-rich diet is associated with positive health outcomes and reduced risk of disease, Alison et al. highlighted the importance of a high-fiber diet in protection against asthma development (Thorburn et al., 2015). Saadeh et al. (2015) observed that consumption of fast food and butter intake were associated with an increased prevalence of asthma symptoms among atopic children. A high-fat diet has been shown to increase airway inflammation in asthma (Wood et al., 2011). Hence, diet could be an environmental factor that influences how probiotics prevent AA.

We monitored systemic symptoms (nasal rubbing and sneezing), cytokines levels, inflammatory cell infiltration, and fecal bacterial composition in a dietary-intervention study of AA (induced by OVA) rats with modulation by *L*. *paracasei*. We discovered that different diets influenced the effect of *L*. *paracasei* on modulating AA. Moreover, a high fiber diet plus *L*. *paracasei* as a rescue medication showed a synergistic effect and led to a decreased frequency of rubbing and sneezes, whereas a high-fat diet exacerbated these symptoms. A reduced serum level of OVA-specific IgE and total counts of inflammatory cells in BALF in the Hfiber group suggested an instinctive response to remission of allergic inflammation, but the group that consumed a high-fat diet showed the opposite results. IgE on the surface of immune cells binds to specific airway allergens followed by IgE cross-linking, cell activation, and release of preformed mediators (Komi and Bjermer, 2019; Salomonsson et al., 2019; Liu et al., 2020). In this regard, the lower IgE level also reflected attenuation of the Th2 immune response and the immune tolerance to allergic antigens (Ciprandi et al., 2015; Strokes and Casale, 2015). AA is characterized by eosinophils accumulation. The decreased number of eosinophils infiltrating the lungs was a response to the inhibition of allergic inflammation by consumption of a high-fiber diet. A high-fat diet induced eosinophil accumulation in the lungs of AA rats.

Th2 cells have crucial roles in AA pathogenesis, and imbalance of Th1/Th2 cells and disturbed balance of Th17/T_*reg*_ cells has been reported in asthma patients. Hence, to discriminate the state of Th1/Th2 and Th17/T_*reg*_ subsets in allergic airways, we measured the levels of IFN-ɤ (Th1 cytokine), IL-4 (Th2 cytokine), IL-17 (Th17 cytokine), and TGF-β (T_*reg*_ cytokine) by ELISA. The Probio group given a high-fiber diet inhibited expression of IL-4 and IL-17 and increased expression of IFN-ɤ, but the group that consumed a high-fat diet had the opposite results. Th2 cytokines are believed to regulate IgE synthesis, and eosinophil numbers/activity are thought to play a significant part in driving AA pathogenesis (Dent et al., 1990; Savelkoul et al., 1991). IL-4 can upregulate expression of chemotactic factors such as eotaxins to promote eosinophil infiltration. IL-17 can drive airway remodeling (Quan et al., 2012). Indeed, our study found that the Hfiber diet assists the *L*. *paracasei* skewed the immune balance toward Th1, with increased IFN-ɤ and conversely decreased IL-4, in commitment with the previous presentation of reduced IgE and fewer eosinophils infiltration. Alteration in the balance of Th1/Th2 cytokines is an essential indicator of functional changes in suppressing the aberrant immune response in allergic diseases (Skapenko and Schulze-Koops, 2007; Cho et al., 2009). Although increased expression of IFN-ɤ has been found in individuals with severe asthma and acute exacerbations (Kumar et al., 2006; Barnes, 2009), there is evidence that probiotics promote IFN-ɤ production to reduce allergic inflammation (Giudice et al., 2010). Higher expression of IFN-ɤ in the Hfiber group with decreased expression of IL-4 compared with that in the Hfat group, and the ratio of IL-4/IFN-ɤ implied skewing of the balance of Th1/Th2 cells. The balance between Th17 and T_*regs*_ cells also suggests a crucial role in asthma pathogenesis (Vroman et al., 2018; Wang et al., 2018; Xu et al., 2018). Various studies have indicated that the homeostatic balance between T_*regs*_ and Th17 cells was altered markedly in asthma exacerbations, and correlates with asthma severity (Zou et al., 2018). TGF-β is produced by T_*regs*_, which have been implicated to inhibit the immune response. They suppress effector T cells of Th1 or Th2 phenotypes. Therefore, increased expression of TGF-β in BALF in the Hfiber group seems rational.

Several studies have associated a changed intestinal microbiota with the etiology of various diseases, and changes in gut microflora composition, in response to diet change (Kau et al., 2011; David et al., 2014). We discovered that consumption of different diet led to alterations in the structure of microbial communities in the gut as well as the composition of intestinal bacteria as evidenced by NMDS (Giloteaus et al., 2016). There was a significant difference in the alpha diversity of microbiota in different diet groups, and the bacterial diversity of the Hfiber group was decreased dramatically.

We revealed that a diet rich in fiber assisted the way that *L*. *paracasei* modified the GM. This finding corroborates the data from a study by Salonen et al. (2014), who reported that a diet high in fiber was associated with decreased bacterial diversity relative to that of other diets. Comparison of 16S rRNA gene sequencing among groups revealed that a high-fiber diet significantly improved GM structure by enriching bacteria of the phylum Bacteroidetes, as reported by Trompette et al. (2014). Consumption of a high-fat diet enhanced the proportions of Proteobacteria and Firmicutes, which indicated the enrichment of specific bacteria in the colon is diet-specific (Filippo et al., 2010; Walker et al., 2011; Campo et al., 2014; Cruz et al., 2014; Ramos-Romero et al., 2018). Phylum Bacteroidetes was increased markedly in the Hfiber group. Salyers and coworkers showed that the bacteria of phylum Bacteroidetes have the functions of carbohydrate fermentation, polysaccharide metabolism, and maintaining the normal physiological function of the intestinal tract (Salyers, 1984). The influence of a high-fat diet led to significant induction of Clostridiales, Lachnospiraceae, Ruminococcaceae, Deltaproteobacteria, and Desulfovibrionaceae. Studies have shown that Clostridiales can be pathogenic, leading to infectious diseases or mild cognitive impairment in mice (Antharam et al., 2013; Vogt et al., 2017). The members of the family Lachnospiraceae are enriched by a low-fiber Western-style diet, are associated with inflammatory diseases, and have been reported to protect against allergy by modulating the immune system (Png et al., 2010; Pascal et al., 2018). The relative abundance of bacteria of the family Ruminococcaceae is lower in IgE-associated eczema compared with that in people not suffering from allergies. Enriched abundance of Deltaproteobacteria is related to AA in mice (Hirota et al., 2019). Moreover, bacteria of the family Desulfovibrionaceae (which are opportunistic pathogens) have been linked to inflammatory diseases of the gut and chronic disorders. Hence, the alterations induced by a high-fat diet suggest inflammation exacerbation.

Our correlation analysis of intestinal bacteria and cytokine expression (section “Correlation Analysis for the Intestinal Microbiota and Cytokine Expression in BALF”) demonstrated that GM alterations might be associated with inflammation during AA. Therefore, *L*. *paracasei*, with the assistance of a high-fiber diet, ameliorated allergy symptoms to a greater extent than use of *L*. *paracasei* alone in an inflammatory-modulation manner. Thus, a high fiber diet seems to support the effect of AA medication as an add-on therapy.

Our study had two main limitations. First, our findings are limited to an AA model induced by OVA; the effect a high-fiber or high-fat diet on an AA sensitized by other antigens (e.g., house dust mite) is not known. Second, associations between the most relevant taxa and a high-fiber diet or high-fat diet, respectively, were not validated by real-time reverse transcription-quantitative PCR.

## Conclusion

We provided new insights into consumption of different diets influenced the effect of *L*. *paracasei* on suppressing AA. In particular, the intestinal microbiota altered by different dietary patterns were associated with allergic inflammation.

## Data Availability Statement

The datasets presented in this study can be found in online repositories. The names of the repository/repositories and accession number(s) can be found in the article/Supplementary Material.

## Ethics Statement

The studies involving human participants were reviewed and approved by the Ethics Committee of Dalian Medical University. The patients/participants provided their written informed consent to participate in this study. The animal study was reviewed and approved by the Animal Ethics Committee of Dalian Medical University.

## Author Contributions

SW conceived and designed the experiments and supervised. AX, JS, and SL performed the experiments. JS and YL analyzed the data. AX wrote the manuscript. SW and LT reviewed the manuscript. All authors have read and given approval to the final version of the manuscript.

## Conflict of Interest

The authors declare that the research was conducted in the absence of any commercial or financial relationships that could be construed as a potential conflict of interest.

## Publisher’s Note

All claims expressed in this article are solely those of the authors and do not necessarily represent those of their affiliated organizations, or those of the publisher, the editors and the reviewers. Any product that may be evaluated in this article, or claim that may be made by its manufacturer, is not guaranteed or endorsed by the publisher.

## Footnotes

1
www.qiime.org

## Supplementary Material

Supplementary Figure 1 The ratio of IL-4/IFN-γ.

Supplementary Figure 2 Gut microbiota comparisons between Con group and Model group.

Supplementary Figure 3 Gut microbiota comparisons between Model group and Probio group.

Supplementary Figure 4 Gut microbiota comparisons between Probio group and Hfat group.

Supplementary Figure 5 Gut microbiota comparisons between Probio group and Hfiber group.

Supplementary Table 1 Diet composition.
