# Supplementary figures and images for: Influence of Diet on the Effect of the Probiotic Lactobacillus paracasei in Rats Suffering From Allergic Asthma
Source: Front Microbiol. 2021 Sep 27;12:737622. doi: 10.3389/fmicb.2021.737622 (PMC8516095; doi:10.3389/fmicb.2021.737622)

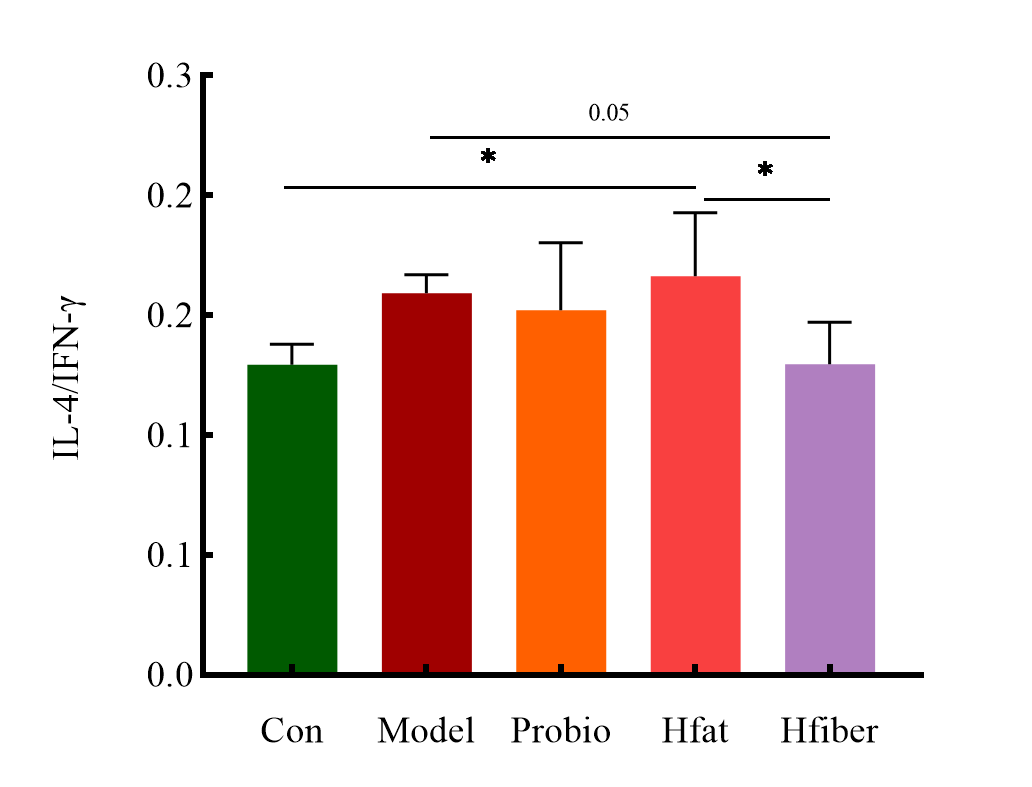

Supplement: Supplementary Figure 1 — The ratio of IL-4/IFN-γ. [file Image_1.tif]

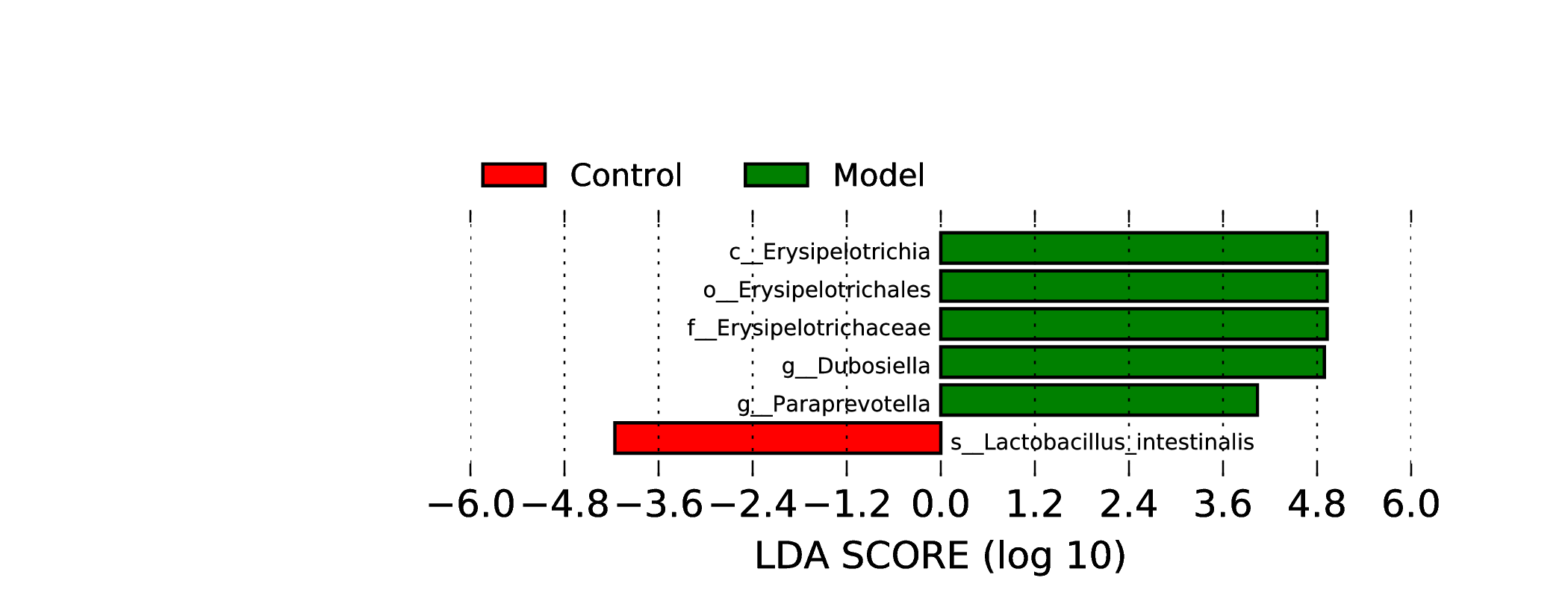

Supplement: Supplementary Figure 2 — Gut microbiota comparisons between Con group and Model group. [file Image_2.png]

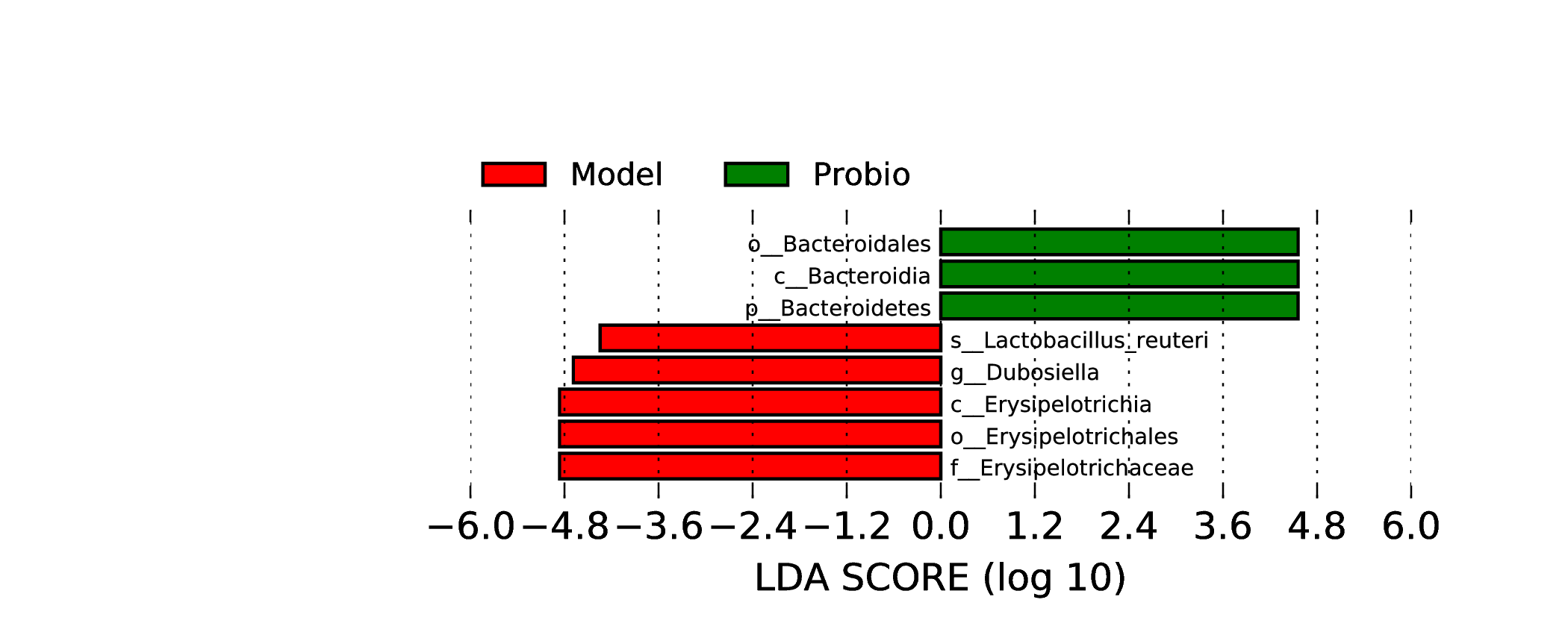

Supplement: Supplementary Figure 3 — Gut microbiota comparisons between Model group and Probio group. [file Image_3.png]

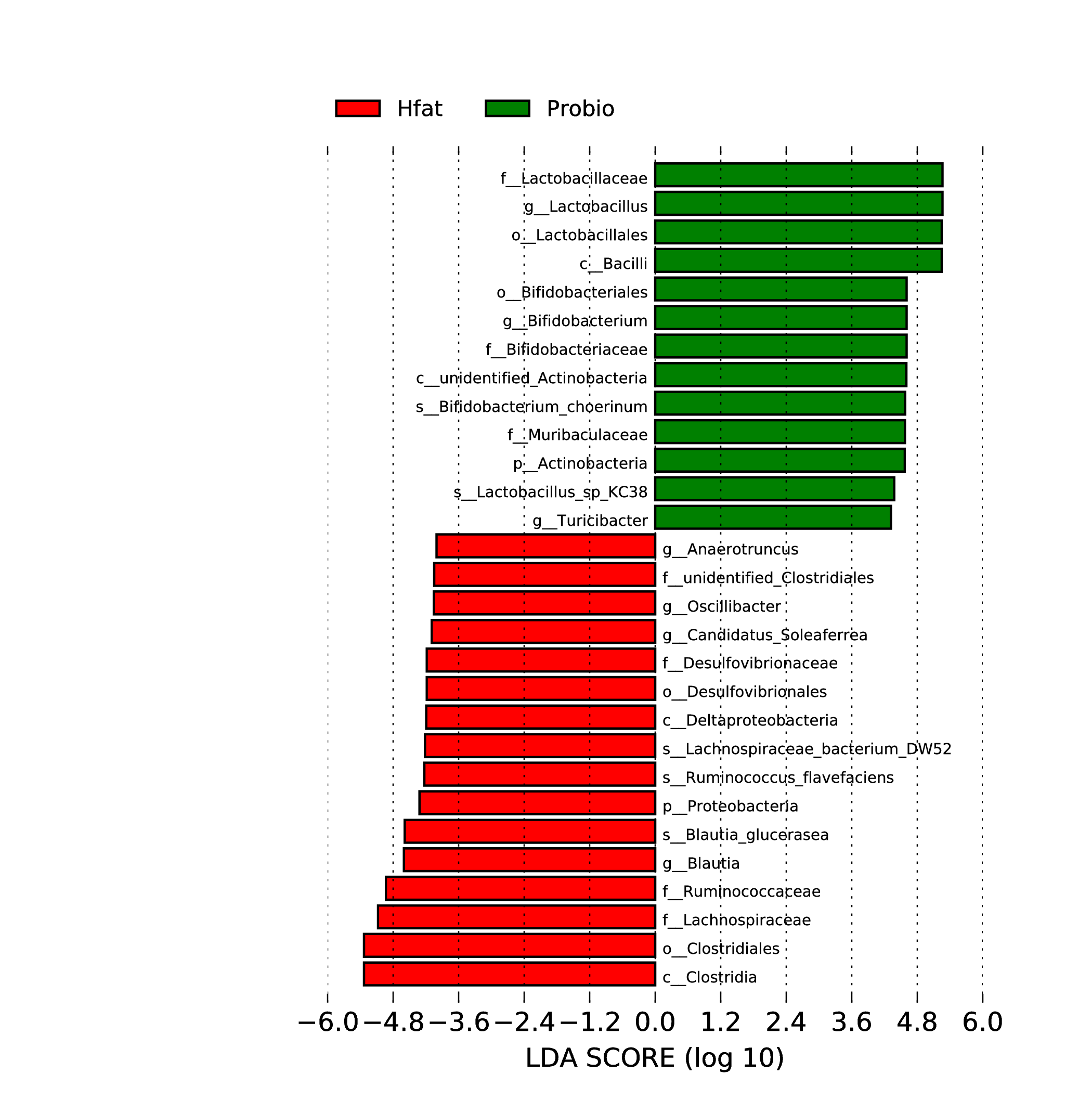

Supplement: Supplementary Figure 4 — Gut microbiota comparisons between Probio group and Hfat group. [file Image_4.png]

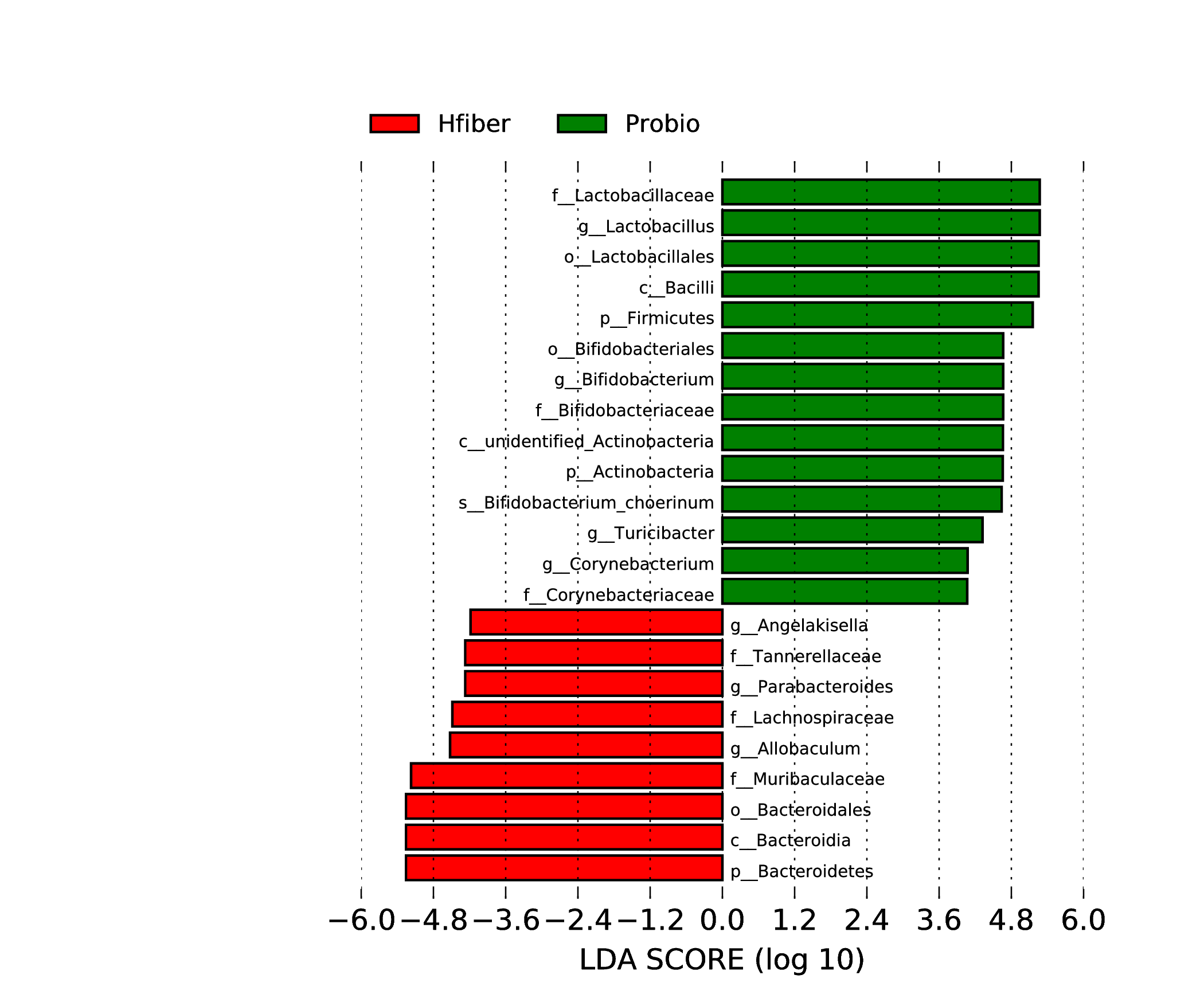

Supplement: Supplementary Figure 5 — Gut microbiota comparisons between Probio group and Hfiber group. [file Image_5.png]
