# Supplementary material for: Influence of Diet on the Effect of the Probiotic Lactobacillus paracasei in Rats Suffering From Allergic Asthma
Source: Front Microbiol. 2021 Sep 27;12:737622. doi: 10.3389/fmicb.2021.737622 (PMC8516095; doi:10.3389/fmicb.2021.737622)
Supplement: Supplementary Table 1 — Diet composition. [file Table_1.DOCX]

**Suppl. Table 1. Diet composition**

| **Diet** | **Ingredient** | **Weight ratio(%)** |
| --- | --- | --- |
| **Basic diet** | Protein | 19.2 |
|  | Fat | 4.6 |
|  | Fiber | 4 |
|  | Carbohydrate | 55.9 |
|  | Water | 18.8 |
|  | Others | 6.3 |
| **High-fat diet** | Protein | 26.0 |
|  | Fat | 35.0 |
|  | Carbohydrate | 26.0 |
| **High-fiber diet** | Protein | 13.8 |
|  | Fat | 3.1 |
|  | Fiber | 30.0 |
|  | Carbohydrate | 38.5 |
|  | Others | 14.6 |
